# Activation of the pentose phosphate pathway by microcurrent stimulation mediates antioxidant effects in inflammation-stimulated macrophages

**DOI:** 10.3389/fphys.2025.1666999

**Published:** 2025-11-14

**Authors:** Mikiko Uemura, Noriaki Maeshige, Atomu Yamaguchi, Xiaoqi Ma, Yunfei Fu, Taketo Inoue, Mami Matsuda, Yuya Nishimura, Tomohisa Hasunuma, Ji Wang, Hiroyo Kondo, Hidemi Fujino

**Affiliations:** 1 Department of Rehabilitation Science, Kobe University Graduate School of Health Sciences, Kobe, Japan; 2 Department of Rehabilitation, Faculty of Health Sciences, Kansai University of Welfare Sciences, Kashiwara-shi, Osaka, Japan; 3 Harvard University T. H Chan School of Public Health, Boston, MA, United States; 4 Assisted Reproductive Technology Center, Okayama University, Okayama, Japan; 5 Graduate School of Science, Technology and Innovation, Kobe University, Kobe, Japan; 6 Engineering Biology Research Center, Kobe University, Kobe, Japan; 7 Department of Toxicology and Sanitary Chemistry, School of Public Health, Capital Medical University, Beijing, China; 8 Department of Nutrition, Faculty of Health and Nutrition, Shubun University, Ichinomiya, Aichi, Japan

**Keywords:** microcurrent stimulation, pentose phosphate pathway (PPP), NADPH, oxidative stress, macrophage, glucose metabolism

## Abstract

**Introduction:**

Excessive inflammatory responses in macrophages lead to increased oxidative stress, and the excessive production of reactive oxygen species (ROS) causes tissue damage, contributing to the development of chronic diseases and tissue deterioration. Therefore, controlling the inflammatory response and ROS production is crucial for human health. Electrical stimulation (ES) has been shown to have antioxidant and anti-inflammatory effects on macrophages. However, the key pathway underlying these effects remains unclear.

**Methods:**

In this study, ES was applied to Lipopolysaccharide (LPS)-stimulated macrophages, and the production of ROS and 8–hydroxy–2′–deoxyguanosine (8-OHdG), inflammatory cytokine expression, and intracellular metabolites were analyzed in a glucose-6-phosphate dehydrogenase (G6PD) knockdown experiment, the rate-limiting enzyme of the Pentose Phosphate Pathway(PPP).

**Results:**

ES significantly increased sedoheptulose 7-phosphate (S7P), an intermediate metabolite in PPP, and reduced ROS and 8-OHdG production and the expression of inflammatory cytokines in LPS-stimulated macrophages. Meanwhile, ES did not exert antioxidant effects in G6PD-knockdown macrophages.

**Discussion:**

These findings indicate that the antioxidant effects of ES are mediated by PPP in LPS-stimulated macrophages.

## Introduction

Excessive inflammation by immune cells is a pathological response contributing to inflammation-related diseases. Macrophages are known to have a pivotal role in this inflammatory process by producing cytokines and chemokines and recruiting other immune cells to the site of inflammation (Turner et al., 2014). In fact, it has been reported that the prolonged macrophage-mediated inflammation causes chronic diseases such as rheumatoid arthritis, chronic ulcers, and atherosclerosis (Krzyszczyk et al., 2018; Luo et al., 2024). Macrophages generate reactive oxygen species (ROS) in response to inflammatory stimuli to attack pathogens (Mittal et al., 2014). However, excessive ROS directly damage intact cells and tissues (Diskin and Palsson-McDermott, 2018). Therefore, ROS are considered major contributors to tissue damage resulting from excessive inflammation.

In inflammatory conditions, macrophages undergo specific metabolic changes known as immunometabolism. Intracellular metabolism shifts to support the production of antioxidants and anti-inflammatory substances as part of a metabolic adaptation to ensure survival. For example, nicotinamide adenine dinucleotide phosphate (NADPH), a metabolite of the pentose phosphate pathway (PPP)—a branch of glycolytic metabolism—is utilized to produce glutathione, a potent antioxidant (Kruger et al., 2011; Yu et al., 2019). Additionally, itaconate, derived from citrate, exerts anti-inflammatory effects by activating NF-E2-related factor 2 (NRF-2) (Zhu et al., 2021; Tannahill et al., 2013). At the same time, however, metabolic products that promote inflammation are also produced. Inflammation disrupts the tricarboxylic acid (TCA) cycle, leading to the accumulation of succinate, which promotes inflammatory cytokine production (Srirussamee et al., 2019). These metabolic shifts highlight the interplay between inflammatory signaling and intracellular metabolic states in macrophages.

Electrical stimulation (ES) has been shown to influence the dynamics and intracellular metabolism of macrophages. Macrophages can be broadly classified into two phenotypes: classically activated pro-inflammatory M1 macrophages and alternatively activated anti-inflammatory M2 macrophages, which play opposing roles in inflammation and tissue repair (Parisi et al., 2018). Previous studies have reported that ES promotes macrophage accumulation, alters the M1/M2 macrophage ratio, and decreases inflammatory cytokine production and oxidative stress (Kao et al., 2013; Meneses et al., 2016; Uemura et al., 2023). However, the dependency of these effects on intracellular metabolism has not been elucidated. In this study, we investigated the antioxidative and anti-inflammatory effects of ES on inflammation-induced macrophages as well as the associated changes in intracellular metabolism through a knockdown experiment of the PPP pathway.

## Materials and methods

### Cells

Bone-marrow derived macrophages (BMDMs) were obtained from bone marrow from 7-week-old male C57BL/6NCrSlc mice and were subsequently cultured in RPMI 1640 medium (186-02155, Fujifilm Wako, Osaka, Japan), with supplementation of 10% fetal bovine serum (FBS), 1% penicillin/streptomycin, 1% L-glutamine, and 25% L929 cell supernatant for a duration of 8 days. BMDMs were plated in 35-mm tissue culture dishes and treated with 100 ng/mL lipopolysaccharide (LPS) for an hour. This study received approval from the Institutional Animal Care and Use Committee and was conducted in compliance with the Kobe University Animal Experimentation Regulations (P210803).

### Electrical stimulation

The culture medium was replaced with FBS-free RPMI 1640 medium, and ES (intensity: 200 μA, frequency: 2 Hz, and duration: 250 ms) was conducted with platinum electrodes immediately after LPS stimulation for 4 h in the 5% CO_2_ incubator at 37 °C.

### Trypan blue staining

Cell viability was analyzed with trypan blue staining to confirm the cell toxicity of ES. The numbers of living and dead cells were counted using a hemocytometer, then cell viabilities were calculated.

### Real time-polymerase chain reaction (RT-PCR)

mRNA was extracted utilizing TRIzol reagent (Thermo Fisher), followed by reverse transcription carried out with the iScript cDNA Synthesis Kit (Bio-Rad, CA, United States) after ES treatment. Subsequently, RT-PCR was performed using specific gene primers under the following cycling conditions: initial denaturation at 95 °C for 3 min, followed by 40 cycles of denaturation at 95 °C for 10 s and annealing/extension at 60 °C for 30 s, using SYBR Green (Invitrogen, MA, United States) and the StepOne™ Real-Time PCR System (Thermo Fisher). mRNA expression levels of *Il-1b*, *Il-6*, *Tnf-a*, *Nrf-2*, and *hypoxanthine phosphoribosyltransferase (Hprt)* were measured. The primer sequences are shown in Supplementary File S1. mRNA expression levels were normalized against *Hprt* as an internal control, and relative expression fold changes were calculated using the 2^−ΔΔCt^ method.

### Detection of ROS

ROS production was assessed by enzyme-linked immunosorbent assay (ELISA) and CellRox Green Reagent (Invitrogen). ELISA was performed to analyze the level of 8-hydroxy-2-deoxyguanosine (8-OHdG) in the cell supernatant. 8-OHdG was measured using a competitive ELISA analysis kit (StressMarq BioSciences, Victoria, BC, Canada) according to the manufacturer’s protocol. Cellrox Green Reagent was introduced to each dish at a concentration of 10 μmol/L, followed by a 30-min incubation period at 37 °C in a 5% CO_2_ incubator. Subsequently, the dishes were rinsed with PBS and subjected to a 15-min incubation at room temperature in 3.7% formaldehyde. After washing with PBS, DAPI (1:1000; Dojindo) was applied, and the cells underwent a 5-min incubation at 37 °C. Following staining, the cells were examined using a fluorescence microscope (Olympus, Tokyo, Japan). The fluorescence intensity of each dish was quantified using ImageJ (Version 1.53u, NIH, MD, United States), as described in Shihan’s study (Shihan et al., 2021), and the results were normalized to those of the control group.

### Metabolite analysis

Each dish was treated with 80% MeOH (water containing 50 μM (+)-10-camphorsulfonic acid, 400 μM L-methionine sulfone, and 400 μM piperazine-1,4-bis(2-ethanesulfonic acid)), followed by a 15-min incubation at −80 °C. Cells were then harvested, centrifuged at 14,000 × g for 5 min, and the resulting supernatant underwent additional centrifugation at 14,000 × g for 90 min at 4 °C, using a 5 kDa cut-off membrane (Merck Millipore, MA, United States) to remove solubilized proteins. The dried metabolites, concentrated by evaporating the aqueous layer extracts with a FreeZone 2.5 Plus freeze-dry system (Labconco, Kansas City, MO), were reconstituted in Milli-Q water. Intracellular metabolites were analyzed using capillary electrophoresis-mass spectrometry (CE/MS, Agilent G7100; MS, Agilent G6224AA LC/MSD TOF; Agilent Technologies, Palo Alto, CA) controlled by MassHunter Workstation Data Acquisition software (Agilent Technologies).

### Gene knockdown

BMDMs were seeded on 35-mm dishes and small interfering RNA (siRNA) for glucose-6-phosphate dehydrogenase (G6PD) was transfected with Lipofectamine RNAiMAX (Thermo Fisher) according to the manufacturer’s instructions. The mRNA expression level of *g6pd* (5′-GCCTCAGTGCTACTAGACATT-3′ and 5′-AGGGTTGGGATAGGAAAA-3′) was measured after 2 days of incubation.

### Statistical analysis

All statistical analyses were performed using EZR (Saitama Medical Center, Jichi Medical University, Saitama, Japan) (Kanda, 2013), which is a graphical user interface for R (The R Foundation for Statistical Computing, Vienna, Austria). Data are expressed as mean ± standard deviation (SD).

For comparisons among three groups, the Tukey–Kramer test was applied. For the analyses of G6PD expression and 8-OHdG levels, homogeneity of variance was first confirmed, and then comparisons between two groups were performed using the Student’s *t*-test. A *p* value of <0.05 was considered statistically significant.

## Results

### LPS or ES did not decrease cell viability

LPS stimulation and combined LPS + ES stimulation did not decrease cell viability (Supplementary Figure S1). These results indicate that neither LPS nor ES exposure to BMDMs caused any cytotoxic effects.

### ES altered glycolytic pathway and enhanced PPP

**FIGURE 1:**
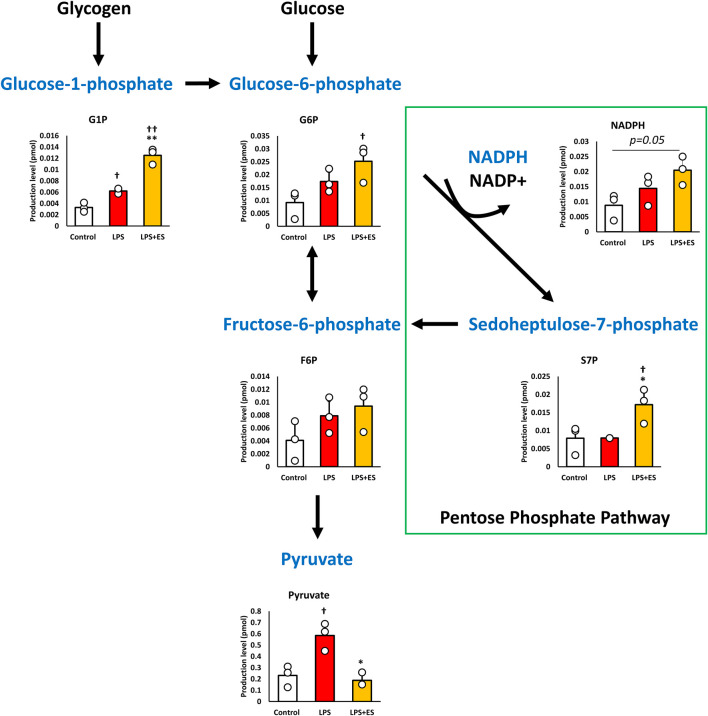
ES enhances glycolytic pathway and PPP. These results present the ratios of the control group. LPS increased G1P, and Pyruvate level (p < 0.05, vs. control group) while ES further increased G6P level (p < 0.05, vs. Control group), but pyruvate level was similar to the control by ES. Moreover, ES increased NADPH level (p = 0.05 vs. Control group) and S7P level (p < 0.05, vs. control and LPS groups). Statistical differences among the groups were analyzed using the Tukey-Kramer test. n = 3 for per group. **p < 0.01, *p < 0.05 vs. LPS group; † † p < 0.01, † p < 0.05 vs. control group.

**FIGURE 2:**
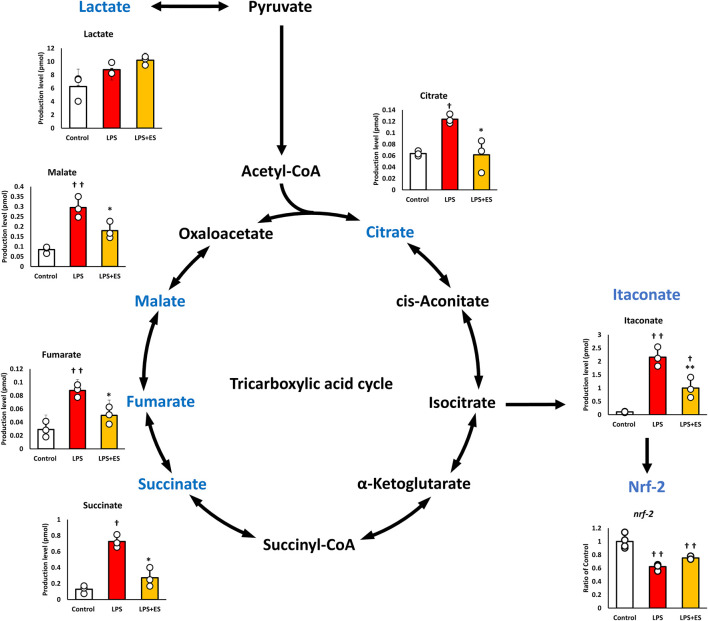
ES attenuates LPS-induced upregulation of TCA cycle metabolites in BMDMs. These results present the ratios of the control group. LPS stimuli significantly increased all metabolites levels while ES significantly decrease these metabolites against LPS group. Itaconate, metabolized from isocitrate was increase by LPS, but ES did not increase. Itaconate activates *Nrf-2* which has anti-inflammatory effect and both LPS and ES did not increase N*rf-2* expression. Statistical differences among the groups were analyzed using the Tukey-Kramer test. n = 3 per group. **p < 0.01, *p < 0.05 vs. LPS group; † † p < 0.01, † p < 0.05 v.s. control group. Data are presented as the mean ± SD.

Alterations in metabolism prompted by LPS exposure and subsequent ES were quantified because of the close relationship between inflammatory responses and intracellular metabolism. Figure 1 shows the intracellular metabolite levels in the glycolytic pathway, and Figure 2 shows the metabolites in TCA cycle. Glycolytic metabolites exhibited an increase following LPS stimulation. However, ES intensified their metabolism beyond that induced by LPS alone, resulting in a significant increase in the production of glucose-1-phosphate (G1P) and glucose-6-phosphate (G6P) compared to the control group. Conversely, the production of pyruvate, derived from fructose-1-phosphate (F6P) metabolism, was elevated by LPS compared to the control group but decreased to levels comparable with the control group after ES. Additionally, the PPP diverges from G6P and follows a pathway back to F6P. Following ES, there was an increase in NADPH and sedoheptulose 7-phosphate (S7P) which are the intermediate metabolites in PPP (p = 0.05 and p < 0.05, respectively).

Pyruvate is metabolized to lactate or acetyl CoA, and the TCA cycle is initiated from acetyl CoA. LPS significantly increased both metabolites of the TCA cycle, but ES decreased the increased production by LPS. Itaconate, which is metabolized from isocitrate, activates NRF-2 and exerts an anti-inflammatory effect, but its level was decreased after ES, even though itaconate level was increased by LPS stimulation. However, *Nrf-2* expression was decreased after both LPS and ES stimulation compared to the control group, suggesting that the anti-inflammatory effect of ES was not mediated by itaconate.

### ES decreased oxidative stress and inflammatory cytokine expression after LPS stimulation

**FIGURE 3:**
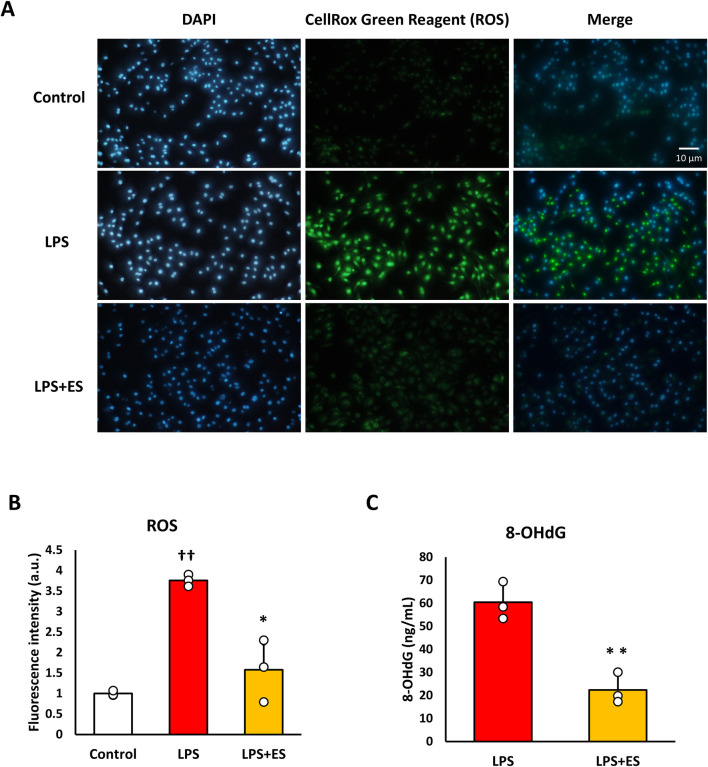
ES suppresses ROS and 8-OHdG production induced by LPS stimulation. **(A)** BMDMs were stained with DAPI or CellRox Green Reagent. LPS-stimulated BMDMs were stained, with fluorescence intensities observed in the control group (higher), the LPS group (middle), and the LPS+ES group (lower). Blue indicates DAPI staining, and green represents ROS. **(B)** The fluorescence intensities for each group were quantified using ImageJ and compared to the control group. The intensity in the LPS group was significantly higher than that of the control group (p < 0.01). The LPS+ES group showed a significant suppression of intensity compared to the LPS group (p < 0.05). **(C)** ES also significantly decreased 8-OHdG level in LPS stimulated macrophages. The 8-OHdG level in the control group was below the detection limit and therefore not displayed. Statistical differences among the groups were analyzed using the Tukey-Kramer test or student-t test. n = 3 per group. **p < 0.01, *p < 0.05 vs. LPS group, † † p < 0.01 vs. control group. Data are presented as the mean ± SD.

**FIGURE 4:**
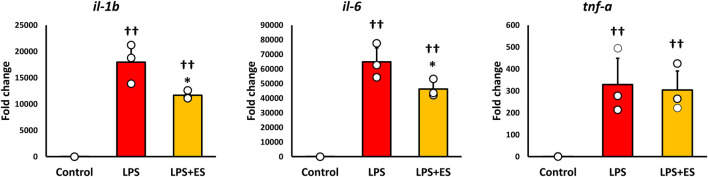
ES suppresses *Il-1b* and *Il-6* expression induced by LPS stimulation. These results represent the ratios in the control group. LPS stimulation significantly increased inflammatory cytokines expression (p < 0.01, vs. control group). ES significantly decreased *Il-1b* and *Il-6* mRNA expressions elevated by LPS (p < 0.05, vs. LPS group), however, *Tnf-a* mRNA expression was not decreased by ES. The statistical differences between these groups were tested by Tukey-Kramer test. n = 3 per group. *p < 0.05, vs. LPS group, and † † p < 0.01, vs. control group. Data are presented as mean ± SD.

ROS was measured to confirm oxidative stress caused by LPS. ROS fluorescence staining and the fluorescent intensity demonstrated that LPS significantly induced ROS production, however, ES reduced LPS-induced increased ROS production to less than half (p < 0.05, vs. LPS group; Figures 3A,B). 8-OHdG, a biomarker of DNA damage caused by ROS, reflects the degree of oxidative damage. As shown in Figure 3C, ES markedly reduced 8-OHdG level compared to that of LPS group (p < 0.01). In addition, ES significantly decreased LPS-induced upregulations of *Il-1b* and *Il-6* mRNA (p < 0.05, vs. LPS group; Figure 4). However, there was no decrease in *Tnf-α* mRNA expression after ES.

### Knockdown of g6pd decreases abolishes the ROS-suppressive effect of ES

NADPH is synthesized when glucose 6-phosphate is transferred to the pentose phosphate pathway and metabolized to 6-phosphogluconolactone, which is regulated by G6PD. LPS stimulation and ES were performed on macrophages with G6PD knocked down to confirm whether the antioxidant and anti-inflammatory effects of ES were due to NADPH production.

**FIGURE 5:**
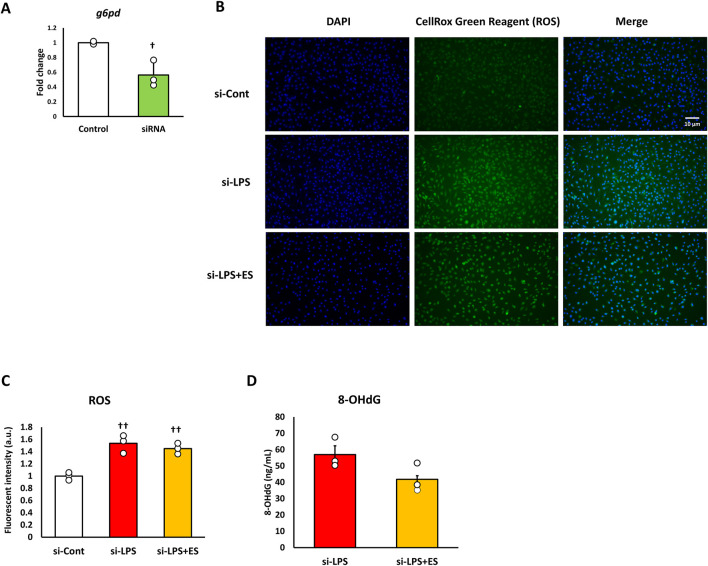
ES does not suppress ROS production or oxidative stress in G6PD-knockdown BMDMs after LPS stimulation. **(A)** *G6pd* mRNA expression was reduced to half that of the control group. This result indicates that *G6pd* was effectively knocked down in siRNA-transfected BMDMs. Statistical differences between the control and siRNA groups were tested using Student’s t-test. n = 3 for per group. † p < 0.05, vs. control group Data are presented as mean ± SD. **(B)** G6PD knockdown-BMDMs were stained with DAPI or CellRox Green Reagent. LPS-stimulated BMDMs were stained, with fluorescence intensities observed in the si-control group (higher), the si-LPS group (middle), and the si-LPS+ES group (lower). **(C)** The fluorescence intensities for each group were quantified using ImageJ and compared to the si-control group. The fluorescent intensity was significantly increased by LPS stimulation, and it was maintained after ES (p < 0.05, vs. si-control group). **(D)** ES also did not reduce the 8-OHdG level in LPS-stimulated macrophages. The 8-OHdG level in the control group was below the detection limit and therefore not displayed. The statistical differences between these groups were tested by Tukey-Kramer test. n = 3 per group. †† p < 0.01, vs. control group. Data are presented as mean ± SD.

**FIGURE 6:**
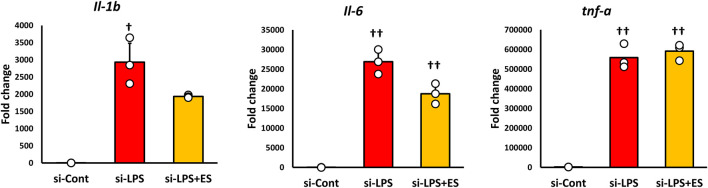
ES does not adequately suppress inflammatory cytokine expression in G6PD-knockdown BMDMs. Inflammatory cytokines expression was analyzed in G6PD-knockdown BMDMs. LPS increased *Il-1b, Il-6,* and *Tnf-a* mRNA expressions (*Il-6*, and *Tnf-a*: p < 0.01, and *Il-1b*: p < 0.05), however, ES did not significantly decrease these cytokines. The statistical differences between these groups were tested by Tukey-Kramer test. n = 3 per group. †† p < 0.01, and † p < 0.05, vs. si-control group. Data are presented as mean ± SD.

After confirming that *g6pd* mRNA expression was reduced to approximately half in siRNA-transfected BMDMs (Figure 5A), LPS stimulation was conducted for 1 h, followed by ES for 4 h. In G6PD-knockdown BMDMs, ES did not reduce the production of ROS or 8-OHdG (Figures 5B–D). Furthermore, ES did not fully exert its anti-inflammatory effects and did not significantly reduce inflammatory cytokine expression in G6PD-knockdown BMDMs (Figure 6).

## Discussion

This study demonstrated that ES exerts antioxidant effects on inflammation-stimulated BMDMs by promoting a shift of glucose metabolism toward the PPP. Inflammatory stimulation of macrophages typically induces ROS production, which has been reported to promote the production of inflammatory cytokines and cause cell damage (Xu et al., 2025). In this study, ES markedly suppressed ROS and 8-OHdG production via PPP activation. These findings suggest the role of ES as an antioxidant therapy and its dependency on the metabolic regulation in PPP.

Macrophages undergo metabolic reprogramming in response to inflammatory stimuli. While some metabolites possess antioxidant and anti-inflammatory properties to support cell survival, some metabolites promote inflammation. To elucidate the mechanism of the antioxidant effects of ES, intracellular metabolites were analyzed in the present study. LPS stimulation increased glycogen metabolite G1P, while ES further elevated G1P and glucose-derived G6P. Additionally, ES increased NADPH level which is an intermediate metabolite in PPP. NADPH contributes to antioxidant defense by facilitating the conversion of oxidized glutathione (GSSG) to its reduced form (GSH), thereby restoring antioxidative capacity in BMDMs (Tannahill et al., 2013; Jiang et al., 2014). NADPH, generated from G6P metabolism, is crucial for cellular survival and has been reported to suppress oxidative stress and reduce cell death (Kruger et al., 2011; Yu et al., 2019). Furthermore, increasing NADPH production exerts antioxidant effects, which suppress inflammatory cytokine production (Cui et al., 2024). NAD(P)H:quinone oxidoreductase 1 (NQO1), derived from NADPH, is also known to exert antioxidant effects on macrophages (Kimura et al., 2018). Therefore, the increase in NADPH production induced by ES observed in this study suggests that ES exerts antioxidant effects on BMDMs under inflammatory stimulation, thereby reducing oxidative stress and suppressing the production of inflammatory cytokines. It is known that ROS can promote inflammatory cytokine production through activation of signaling pathways such as Nuclear factor-κB (NF-κB) and Mitogen-activated Protein Kinase (MAPK) (Canton et al., 2021). However, in the present study, the relationship between ROS suppression by ES and these specific pathways was not directly investigated.

In this study, the G6P and S7P level also increased in response to ES. Previous studies have reported that microcurrent stimulation enhances glucokinase (Gck) activity, which facilitates the conversion of glucose to G6P (Luo et al., 2021). The increase in G6P levels observed in the present study may also reflect enhanced Gck activity induced by ES. Since both S7P and NADPH are metabolic products of the PPP in mammals, these findings suggest that ES shifts glycolytic metabolism toward upregulation of the PPP. S7P is eventually metabolized to F6P, which typically proceeds through glycolysis to pyruvate and the TCA cycle (Stincone et al., 2015). However, under conditions where cells require NADPH production, F6P is reversibly converted to G6P, thereby forming the pentose phosphate cycle (Mailloux et al., 2007; Ting et al., 2023). Although ES decreased pyruvate levels, lactate levels remained comparable between LPS and LPS+ES groups. When the PPP is activated, continuous lactate production from pyruvate sustains NAD^+^ regeneration required for maintaining glycolytic flux (Feron, 2009). Thus, decreased pyruvate but maintained lactate levels indicate activation of the PPP and glycolysis under ES stimulation.

In the present study, the role of the PPP in the antioxidant effects of ES was evaluated by knocking down G6PD, the rate-limiting enzyme for PPP entry, in LPS-stimulated macrophages. In G6PD-knockdown BMDMs, ES did not inhibit the production of ROS and oxidative stress compared to LPS stimulation. These results suggest that the antioxidant effects of ES on LPS-stimulated macrophages are mediated through the enhancement of the PPP. Previous reports indicate that G6PD overexpression in macrophages reduces ROS production in response to inflammation, while inhibition of G6PD activity weakens cell resistance to infection, thereby increasing cell death (Bermudez-Munoz et al., 2020; Zamani et al., 2019). The present study suggests that knockdown of G6PD hindered the transition to the PPP, thereby attenuating the antioxidant effects of ES in LPS-stimulated BMDMs. Inflammatory cytokine production is suppressed by inhibiting oxidative stress, and ES reduced the expression of IL-1β and IL-6 in LPS-stimulated BMDMs. In G6PD knockdown BMDMs, although no significant differences were observed, the expression levels of IL-1β and IL-6 were lower in the LPS+ES group compared to the LPS group. This indicates that ES retained a partial anti-inflammatory effect even in G6PD knockdown BMDMs. LPS stimulation promotes ROS production, which induces nuclear translocation of NF-kB and the expression of inflammatory cytokines (Sharif et al., 2007). At the same time, LPS also activates the Toll-like receptor 4 (TLR4)–MyD88–NF-κB signaling pathway to induce inflammatory cytokines production independently of ROS (Liu et al., 2017). Therefore, even under G6PD-knockdown conditions in which ROS production was maintained, ES partially suppressed cytokine expression. This suggests that ES may exert anti-inflammatory effects not only through the reduction of oxidative stress but also via modulation of ROS-independent inflammatory signaling pathways.

In the present study, ES did not suppress TNF-α expression. Similar findings have been reported, indicating that elevated GSH levels do not influence TNF-α expression (Chang et al., 2011; Barquilha et al., 2023; Zhang et al., 2023). Zhang et al. reported that dimethyl fumarate, which increases GSH levels, suppresses IL-6 expression by inhibiting IκB-ζ but does not suppress TNF-α expression (Zhang et al., 2023). Under LPS stimulation, TNF-α expression in macrophages is induced by NF-κB activation; however, IκB-ζ is known to play a role in the switch from TNF-α production to enhanced IL-6 production (Yamamoto et al., 2004). These observations suggest that the antioxidant effects of ES mediated by NADPH may not directly influence TNF-α production.

LPS stimulation disrupts the TCA cycle, significantly increasing the production of itaconate and succinate, two metabolites associated with inflammation. ES reduced the production of both metabolites compared to LPS stimulation alone. Itaconate, a metabolite known to activate NRF-2 and suppress inflammatory cytokines (Yu et al., 2019; Zhu et al., 2021); however, ES increased neither itaconate nor NRF-2 expression in the present study. Succinate, which promotes inflammation and induces IL-1β expression (Tannahill et al., 2013), also decreased after ES. These findings suggest that the suppression of IL-1β by ES may partly result from reduced succinate accumulation.

This study demonstrated that microcurrent ES exerts antioxidant effects on inflammation-stimulated macrophages via PPP activation. Based on these results, ES could have therapeutic potential for chronic inflammatory conditions as it could reduce excessive inflammatory responses by modulating macrophage metabolism and promoting antioxidant effects. Although the present study revealed an increase in G1P levels following ES, the underlying mechanism remains unclear. Further studies are needed to clarify whether this change reflects altered glycogen metabolism or secondary regulation associated with PPP activation. Moreover, to further clarify the role of NADPH in the antioxidant effects of ES, future studies will include extracellular NADPH supplementation to examine its contribution to ES-induced protective mechanisms.

## Conclusion

ES exerts antioxidant effects by enhancing the PPP in inflammatory-stimulated macrophages.

## Data Availability

The original contributions presented in the study are included in the article/Supplementary Material, further inquiries can be directed to the corresponding author.

## Supplementary material

SUPPLEMENTARY FIGURE S1 ES did not decrease cell viability. LPS stimulation and combined LPS + ES stimulation did not decrease cell viability compared with the control group. The statistical differences between these groups were tested by Tukey-Kramer test. n = 4 per group. Data are presented as mean ± SD.

## References

[B1] Barquilha G. Santos C. M. Cacula K. G. Santos V. C. Polotow T. G. Vasconcellos C. V. (2023). Fish oil supplementation improves the repeated-bout effect and redox balance in 20–30-year-old men submitted to strength training. Nutrients 15, 1708. 10.3390/nu15071708 37049548 PMC10096819

[B2] Bermudez-Munoz J. M. Celeya A. M. Pechero S. H. Wang J. Serrano M. Nieto I. V. (2020). G6PD overexpression protects from oxidative stress and age-related hearing loss. Aging Cell 19, e13275. 10.1111/acel.13275 33222382 PMC7744953

[B3] Canton M. Sánchez-Rodríguez R. Spera I. Venegas F. C. Favia M. Viola A. (2021). Reactive oxygen species in macrophages: sources and targets. Front. Immunol. 30. 10.3389/fimmu.2021.734229 34659222 PMC8515906

[B4] Chang C. C. Chang C. Y. Wu Y. T. Huang J. P. Yen T. H. Hung L. M. (2011). Resveratrol retards progression of diabetic nephropathy through modulations of oxidative stress, proinflammatory cytokines, and AMP-activated protein kinase. J. Biomed. Sci. 18, 47. 10.1186/1423-0127-18-47 21699681 PMC3150248

[B5] Cui Y. Li Z. Ni L. Yu S. Shan X. Hu P. (2024). Induction of MTHFD2 in macrophages inhibits reactive oxygen species-mediated NF-κB activation and protects against inflammatory responses. J. Immunol. 212, 1345–1356. 10.4049/jimmunol.2300209 38407485

[B6] Diskin C. Palsson-McDermott E. M. (2018). Metabolic modulation in macrophage effector function. Front. Immunol. 9, 270. 10.3389/fimmu.2018.00270 29520272 PMC5827535

[B7] Feron O. (2009). Pyruvate into lactate and back: from the Warburg effect to symbiotic energy fuel exchange in cancer cells. Radiother. Oncol. 92 (3), 329–333. 10.1016/j.radonc.2009.06.025 19604589

[B8] Jiang P. Du W. Wu M. (2014). Regulation of the pentose phosphate pathway in cancer. Protein Cell 5, 592–602. 10.1007/s13238-014-0082-8 25015087 PMC4112277

[B9] Kanda Y. (2013). Investigation of the freely available easy-to-use software ‘EZR’ for medical statistics. Bone Marrow Transpl. 48 (3), 452–458. 10.1038/bmt.2012.244 23208313 PMC3590441

[B10] Kao C. H. Chen J. J. J. Hsu Y. M. Bau D. T. Yao C. H. Chen Y. S. (2013). High-frequency electrical stimulation can be a complementary therapy to promote nerve regeneration in diabetic rats. PLoS One 8, e79078. 10.1371/journal.pone.0079078 24265744 PMC3827114

[B11] Kimura A. Kitajima M. Nishida K. Serada S. Fujimoto M. Naka T. (2018). NQO1 inhibits the TLR-dependent production of selective cytokines by promoting IκB-ζ degradation. J. Exp. Med. 215, 2197–2209. 10.1084/jem.20172024 29934320 PMC6080903

[B12] Kruger A. Gruning N. M. Wamelink M. M. C. Erich M. Kirpy A. Parkhomchuk D. (2011). The pentose phosphate pathway is a metabolic redox sensor and regulates transcription during the antioxidant response. Antioxid. Redox Signal 15, 311–324. 10.1089/ars.2010.3797 21348809

[B13] Krzyszczyk P. Schloss R. Palmer A. Berthiaume F. (2018). The role of macrophages in acute and chronic wound healing and interventions to promote pro-wound healing phenotypes. Front. Physiol. 9, 419. 10.3389/fphys.2018.00419 29765329 PMC5938667

[B14] Liu T. Zhang L. Joo D. Sun S. C. (2017). NF-κB signaling in inflammation. Signal Transduct. Target Ther. 2, 17023. 10.1038/sigtrans.2017.23 29158945 PMC5661633

[B15] Luo Y. C. Huang S. H. Pathak N. Chuang Y. H. Yang J. M. (2021). An integrated systematic approach for investigating microcurrent electrical nerve stimulation (MENS) efficacy in STZ-induced diabetes mellitus. Life Sci. 279, 119650. 10.1016/j.lfs.2021.119650 34048807

[B16] Luo M. Zhao F. Cheng G. Su M. Wang Y. (2024). Macrophage polarization: an important role in inflammatory diseases. Front. Immunol. 15, 1352946. 10.3389/fimmu.2024.1352946 38660308 PMC11039887

[B17] Mailloux R. J. Bériault R. Lemire J. Singh R. Chénier D. R. Hamel R. D. (2007). The tricarboxylic acid cycle, an ancient metabolic network with a novel twist. PLoS One 2, e690. 10.1371/journal.pone.0000690 17668068 PMC1930152

[B18] Meneses G. Bautista M. Florentino A. Diaz G. Acero G. Besedovsky H. (2016). Electric stimulation of the vagus nerve reduced mouse neuroinflammation induced by lipopolysaccharide. J. Inflamm. 13, 33. 10.1186/s12950-016-0140-5 27807399 PMC5086408

[B19] Mittal M. Siddiqui M. R. Tran K. Reddy S. P. Malik A. B. (2014). Reactive oxygen species in inflammation and tissue injury. Antioxid. Redox Signal 20, 1126–1167. 10.1089/ars.2012.5149 23991888 PMC3929010

[B20] Parisi L. Gini E. Baci D. Tremolati M. Fanuli M. Bassani B. (2018). Macrophage polarization in chronic inflammatory diseases: killers or builders? J. Immunol. Res. 2018, 8917804. 10.1155/2018/8917804 29507865 PMC5821995

[B21] Sharif O. Bolshakov V. N. Raines S. Newham P. Perkins N. D. (2007). Transcriptional profiling of the LPS induced NF-kappaB response in macrophages. BMC Immunol. 8, 1. 10.1186/1471-2172-8-1 17222336 PMC1781469

[B22] Shihan M. H. Novo S. G. Le Marchand S. J. Wang Y. Duncan M. K. (2021). A simple method for quantitating confocal fluorescent images. Biochem. Biophys. Rep. 25, 100916. 10.1016/j.bbrep.2021.100916 33553685 PMC7856428

[B23] Srirussamee K. Mobini S. Cassidy N. J. Cartmell S. H. (2019). Direct electrical stimulation enhances osteogenesis by inducing Bmp2 and Spp1 expressions from macrophages and preosteoblasts. Biotechnol. Bioeng. 116, 3421–3432. 10.1002/bit.27142 31429922 PMC6899728

[B24] Stincone A. Prigione A. Cramer T. Wamelink M. M. C. Campbell K. Cheung E. (2015). The return of metabolism: biochemistry and physiology of the pentose phosphate pathway. Biol. Rev. Camb Philos. Soc. 90, 927–963. 10.1111/brv.12140 25243985 PMC4470864

[B25] Tannahill G. M. Curtis A. M. Adamik J. Palsson-McDermott E. M. McGettrick A. F. Goel G. (2013). Succinate is an inflammatory signal that induces IL-1β through HIF-1α. Nature 496, 238–242. 10.1038/nature11986 23535595 PMC4031686

[B26] Ting K. K. Jongstra-Bilen J. Cybulsky M. (2023). The multi-faceted role of NADPH in regulating inflammation in activated myeloid cells. Front. Immunol. 14, 1328484. 10.3389/fimmu.2023.1328484 38106413 PMC10722250

[B27] Turner M. D. Nedjai B. Hurst T. Pennington D. J. (2014). Cytokines and chemokines: at the crossroads of cell signalling and inflammatory disease. Biochim. Biophys. Acta 1843, 2563–2582. 10.1016/j.bbamcr.2014.05.014 24892271

[B28] Uemura M. Maeshige N. Yamaguchi A. Xiaoqi M. Matuda M. Nishimura Y. (2023). Electrical stimulation facilitates NADPH production in pentose phosphate pathway and exerts an anti-inflammatory effect in macrophages. Sci. Rep. 13, 17819. 10.1038/s41598-023-44886-x 37857669 PMC10587116

[B29] Xu X. Pang Y. Fan X. (2025). Mitochondria in oxidative stress, inflammation and aging: from mechanisms to therapeutic advances. Signal Transduct. Target Ther. 10, 190. 10.1038/s41392-025-02253-4 40500258 PMC12159213

[B30] Yamamoto M. Yamazaki S. Uematsu S. Sato S. Hemmi H. Hoshino K. (2004). Regulation of Toll/IL-1-receptor-mediated gene expression by the inducible nuclear protein IkappaBzeta. Nature 430, 218–222. 10.1038/nature02738 15241416

[B31] Yu X. H. Zhang D. W. Cheng X. L. Tang C. K. (2019). Itaconate: an emerging determinant of inflammation in activated macrophages. Immunol. Cell Biol. 97, 134–141. 10.1111/imcb.12218 30428148

[B32] Zamani S. Hoseini A. Z. Namin A. M. (2019). Glucose-6-phosphate dehydrogenase (G6PD) activity can modulate macrophage response to Leishmania major infection. Int. Immunopharmacol. 69, 178–183. 10.1016/j.intimp.2019.01.028 30716588

[B33] Zhang Y. Tang J. Zhou Y. Xiao Q. Chen Q. Wang H. (2023). Short-term exposure to dimethyl fumarate (DMF) inhibits LPS-induced IκBζ expression in macrophages. Front. Pharmacol. 14, 1114897. 10.3389/fphar.2023.1114897 36817140 PMC9929133

[B34] Zhu X. Guo Y. Liu Z. Yang J. Tang H. Wang Y. (2021). Itaconic acid exerts anti-inflammatory and antibacterial effects via promoting pentose phosphate pathway to produce ROS. Sci. Rep. 11, 18173. 10.1038/s41598-021-97352-x 34518559 PMC8438069

