# Supplementary material for: Activation of the pentose phosphate pathway by microcurrent stimulation mediates antioxidant effects in inflammation-stimulated macrophages
Source: Front Physiol. 2025 Nov 14;16:1666999. doi: 10.3389/fphys.2025.1666999 (PMC12660068; doi:10.3389/fphys.2025.1666999)
Supplement: Supplementary file 1 [file Table1.docx]

Supplementary file 1. Primer sequences

| Primer | Sequences |
| --- | --- |
| *Hprt* – Forward | 5’-GCTTGCTGGTGAAAAGGACCTCTCGAAG-3’ |
| *Hprt* – Reverse | 5'-CCCTGAAGTACTCATTATAGTCAAGGGCAT-3’ |
| *Il-1β* – Forward | 5’-ACAAAGCCAGAGTCCTTCAGAGAG-3’ |
| *Il-1β* – Reverse | 5’-TTGGATGGTCTTGGTCCTTAGCCA-3’ |
| *Il-6* – Forward | 5’-CCTATGTCTCAGCCTCTTCT-3’ |
| *Il-6* - Reverse | 5’-GGGAACTTCTCATCCCTTTG-3’ |
| *Tnf-α* – Forward | 5’-CAGCATAGAGCAGGACATGGAG-3’ |
| *Tnf-α* - Reverse | 5’-GAACAGCGGTAGTATCAGCCAG-3’ |
| *Nrf-2 -* Reverse | 5’-GAACAGCGGTAGTATCAGCCAG-3’ |
| *Nrf-2 -* Forward | 5’-CAGCATAGAGCAGGACATGGAG-3’ |
| *G6pd* - Reverse | 5’-AGGGTTGGGATAGGAAAA-3’ |
| *G6pd* - Forward | 5’-GCCTCAGTGCTACTAGACATT-3’ |
