# Supplementary figures and images for: Activation of the pentose phosphate pathway by microcurrent stimulation mediates antioxidant effects in inflammation-stimulated macrophages
Source: Front Physiol. 2025 Nov 14;16:1666999. doi: 10.3389/fphys.2025.1666999 (PMC12660068; doi:10.3389/fphys.2025.1666999)

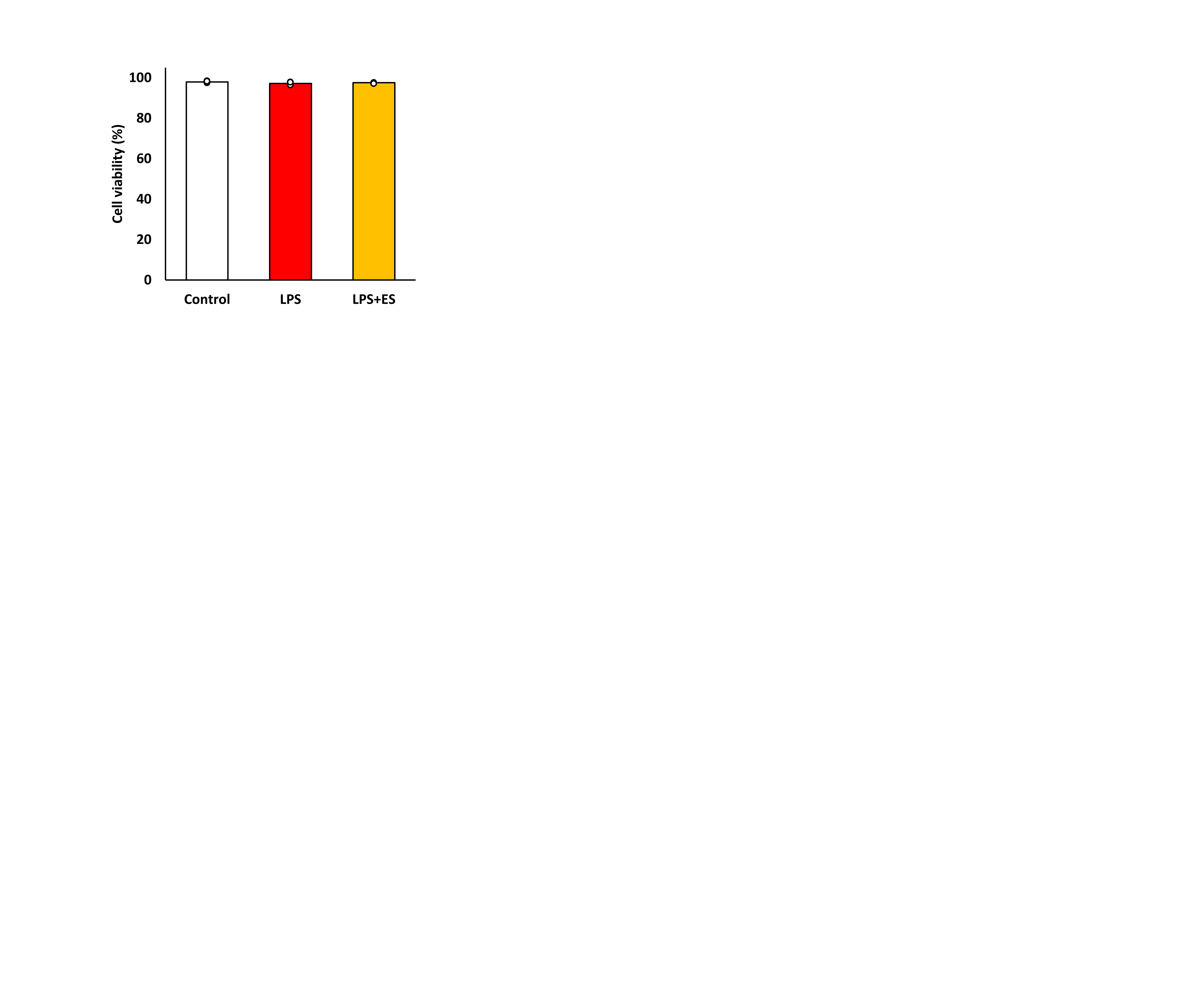

Supplement: Supplementary file 2 [file Image1.tif]
